# 高效液相色谱-三重四极杆质谱法测定环境空气中40种醛酮类化合物

**DOI:** 10.3724/SP.J.1123.2025.04028

**Published:** 2026-06-08

**Authors:** Guanghui LI, Shuo DENG, Qinqin LI, Chunlin ZHANG, Yunfeng LIU, Bin JIANG, Qian YAO, Hao WANG, Boguang WANG

**Affiliations:** 暨南大学环境与气候学院，广东 广州 511443; College of Environmental and Climate，Jinan University，Guangzhou 511443，China

**Keywords:** 醛酮类化合物, 环境空气, 高效液相色谱-三重四极杆质谱, carbonyl compounds （CCs）, ambient air, high performance liquid chromatography-triple quadrupole mass spectrometry （HPLC-MS/MS）

## Abstract

醛酮类化合物（CCs）是环境空气中非常重要的一类含氧挥发性有机物，在大气光化学反应、臭氧生成、二次有机气溶胶形成等过程中具有重要作用。本研究建立了使用高效液相色谱-三重四极杆质谱单次进样同时测定环境空气中40种CCs的分析方法。优化了样品采集、前处理和仪器分析条件。环境空气样品使用2，4-二硝基苯肼（DNPH）管采集（采样流量为1 L/min，采样时长为4 h），采用乙腈洗脱、定容（5 mL），经0.22 μm滤膜过滤后进样分析。采用Sepax BR-C18色谱柱（250 mm×4.6 mm， 5 μm）进行分离，以1.0 mmol/L乙酸铵水溶液-乙腈为流动相进行梯度洗脱，柱温50 ℃，进样量5 μL，采用负离子电喷雾离子源（ESI）进行多反应监测（MRM）扫描。结果表明，40种CCs在2.5~200 μg/L范围内线性关系良好（*R*^2^≥0.997 8），方法检出限为0.001~0.03 μg/m^3^，方法定量限为0.005~0.12 μg/m^3^，低、中、高3个水平下的空白加标回收率为75.2%~119.0%，相对标准偏差为0.4%~4.2%。将其应用于珠三角实际样品的分析，除糠醛和异佛尔酮外，其余38种CCs均可有效检出，其中甲醛、乙醛和壬醛的含量在各观测点均较高；整体上，低分子质量单羰基化合物（LMW-MCs）的含量略高于高分子质量单羰基化合物（HMW-MCs），远高于二羰基化合物（DCs）。本方法简便快速，具有良好的灵敏度、精准度和分离度，有效解决了现有方法中DCs和HMW-MCs种类少、检测困难等问题，适用于环境空气中CCs的检测。

## Abstract (en)

Carbonyl compounds （CCs） are a pivotal class of oxygenated volatile organic compounds in ambient air， which are mainly derived from natural emissions， primary emissions from anthropogenic sources， and secondary formation due to atmospheric oxidation. Various classes of CCs have different impacts on human health， and play critical roles in atmospheric photochemical reactions， ozone production， and the formation of secondary organic aerosols. At present， the number of CCs in ambient air determined by traditional methods is limited， especially for high molecular weight monocarbonyl compounds （HMW-MCs） and dicarbonyl compounds （DCs）. If more CCs can be accurately determined， it will be beneficial for the study of atmospheric oxidation mechanisms and the formulation of pollution control strategies.In this study， an analytical method was developed that enables simultaneous determination of the 40 CCs in ambient air with a single injection， utilizing high-performance liquid chromatography-triple quadrupole mass spectrometry. First， the 16 CCs-2，4-dinitrophenylhydrazine （DNPH）-derivatized single standards were prepared using purified DNPH crystals in the laboratory. Second， the remaining 24 commercial CCs-DNPH-derivatized hybrid standards were purchased. Finally， all the above standards were mixed in a certain proportion to prepare the 40 CCs-DNPH-derivatized hybrid standards for later use. The sample collection procedures （sampling flow rate and sampling duration）， the pretreatment methods （elution volume and constant volume）， the chromatographic analysis conditions （chromatographic column， the concentration of ammonium acetate， etc.） and the mass spectrometry analysis conditions （ion source parameters， fragmentation voltage， collision energy， etc.） were optimized. Ambient air samples were collected using DNPH cartridges （at a flow rate of 1 L/min， for 4 h）， eluted with 4 mL of acetonitrile and diluted to a final volume of 5 mL （the acetonitrile naturally flowed through the DNPH cartridges in the opposite direction to the sampling direction） in a vacuum drying chamber， and then filtered through a 0.22 μm organic filter membrane and transferred to 1.5 mL brown sample bottles prior to instrumental analysis. Chromatographic separation was performed using a Sepax BR-C18 column （250 mm×4.6 mm， 5 μm） with mobile phases consisting of 1.0 mmol/L ammonium aqueous solution and acetonitrile under gradient elution conditions. The analysis was performed at a column temperature of 50 ℃ with an injection volume of 5 μL. The target analytes were identified using negative electrospray ionization （ESI^-^） and multiple reaction monitoring （MRM） modes and quantified using the external standard method. The results showed that the 40 CCs exhibited good linearities within the mass concentration range of 2.5–200 μg/L， with the determination coefficients （*R*^2^） exceeding 0.997 8. The method detection limits and method quantification limits were established within the ranges of 0.001–0.03 μg/m^3^ and 0.005–0.12 μg/m^3^， respectively. The blank spiked recoveries of the target analytes at low， middle， and high concentration levels （0.05， 0.1， and 0.2 μg） ranged from 75.2% to 119.0%， while the relative standard deviations （RSDs， *n*=7） ranged from 0.4% to 4.2%. This analytical method was applied to the monitoring of ambient samples in the Pearl River Delta region. Except for 2-furaldehyde and isophorone， 38 CCs were effectively detected. Among these， formaldehyde， acetaldehyde and nonanal consistently showed higher concentrations across all sampling sites. Overall， the concentrations of low molecular weight monocarbonyl compounds （LMW-MCs） were moderately higher than those of HMW-MCs， and substantially higher than those of DCs. Compared with conventional methods， this method effectively overcame the limitations in existing approaches regarding the limited variety and detection difficulties of DCs and HMW-MCs. Additionally， it also improved the chromatographic resolution of target analytes which have similar retention times and the same ion pairs. Overall， this method was simple and rapid， offering excellent sensitivity， precision， and chromatographic resolution. It was suitable for the determination of the abovementioned 40 CCs in ambient air．

醛酮类化合物（CCs）是环境空气中普遍存在但非常重要的一类含氧挥发性有机物（OVOCs）^［1，2］^，具有强烈的刺激性气味，对人体健康有不同程度的危害，如刺激皮肤、损伤呼吸系统、致癌等^［3-5］^，主要来源于自然排放（植物等）、人为一次排放（机动车尾气、生物质燃烧、工业生产等）及大气氧化二次生成^［1，4，6-8］^。CCs是大气光化学反应的重要中间产物、光化学烟雾的主要成分，也是自由基、过氧酰基硝酸酯、臭氧和二次有机气溶胶（SOA）的重要前体物^［3，4］^。

环境空气中CCs具有含量范围跨度大、反应活性高、性质不稳定等特点^［8］^，因此对样品采集和仪器分析的要求较高。目前，环境空气中CCs的离线分析方法主要有分光光度法^［9］^、毛细管电泳-紫外法^［10］^、气相色谱-质谱联用法（GC-MS）^［1，11］^、2，4-二硝基苯肼（DNPH）管采样-高效液相色谱-紫外或二极管阵列检测法（HPLC-UV/DAD）^［6，9，12，13］^、DNPH管采样-高效液相色谱-质谱联用法（HPLC-MS）^［9，14-17］^等。自2014年我国颁布《环境空气 醛、酮类化合物的测定 高效液相色谱法》（HJ 683-2014）^［18］^以来，DNPH管采样-HPLC-UV/DAD得到了广泛应用；采样时CCs与采样管内涂覆的DNPH发生反应，生成化学性质稳定的衍生物（2，4-二硝基苯腙），使其具有高效、灵敏、选择性好等优点^［6，19，20］^。但HJ 683-2014标准中的方法仅能分析13种CCs，包括9种C_1_~C_4_低分子质量的单羰基化合物（LMW-MCs），4种C_5_以上高分子质量单羰基化合物（HMW-MCs）（主要来自二次转化，环境含量偏低^［7］^），未包括二羰基化合物（DCs）（反应活性较强、环境含量较高^［21］^）。UV/DAD对甲醛、丙烯醛、丁烯醛等的准确定量易受环境影响^［6，22］^。此外，环境空气中许多CCs尤其HMW-MCs的含量偏低，在UV/DAD上响应极低、不能有效检出^［16］^。一旦分析CCs的种类增多，仅依靠优化色谱条件难以实现全部目标物的有效分离。DNPH管采样-HPLC-MS不仅具备DNPH管采样-HPLC-UV/DAD的全部优点，相较于UV/DAD，MS具有更高的灵敏度。可通过母离子、子离子的选择性扫描，更能准确定性、定量，为实现单次进样同时分析更多种CCs提供了可能。

尽管DCs和HMW-MCs对大气氧化性和SOA形成有重要贡献^［3，4，7，21］^，但目前HPLC-MS能测定的种类依然较少^［13，14，16，17］^。为了实现准确测定环境空气中更多种类的CCs，本研究参考现有DNPH管采样-HPLC-MS的研究成果^［4，13，14］^，基于DNPH管采样，乙腈洗脱、定容，采用高效液相色谱-电喷雾离子源-三重四极杆质谱（HPLC-ESI-MS/MS）分析，建立了单次进样同时测定环境空气中40种CCs（包括9种LMW-MCs、29种HMW-MCs和2种DCs）的分析方法，并成功应用于实际样品的分析，为环境空气中CCs的精准测定提供技术支撑。

## 1 实验部分

### 1.1 仪器、试剂与材料

1260-6460高效液相色谱-三重四极杆质谱仪（美国Agilent公司）；GENIUS-NM32LA氮气发生器（英国PEAK公司）；HL-2数显双气路恒流采样泵（北京劳保所）；加热板（日本Asone公司）；LC-DCY-24GP氮吹仪（上海力辰仪器科技有限公司）；微量注射器（瑞士Hamilton公司）；真空干燥器（深圳赛尔玛生物技术有限公司）；BT25S十万分之一天平（德国Sartorius公司）；DNPH管、除臭氧小柱（美国Waters公司）；0.22 μm石英过滤纸（美国Supelco公司）。

市售24种CCs-DNPH衍生混合标准液（以CCs计质量浓度均为100 mg/L，美国Scotty Gases公司）；16种CCs未衍生单标（纯度>99.8%，上海阿拉丁试剂有限公司）；DNPH晶体（纯度为99.5%，美国Fluka公司）；乙腈、甲醇和异丙醇（LC-MS级，德国Merck公司）；乙醇、浓硫酸和乙酸铵（广州化学试剂厂）；超纯水（电阻率18.25 MΩ·cm）；高纯氮气（纯度>99.999%，广州广气气体有限公司）。

### 1.2 2，4-二硝基苯肼晶体的提纯

市售DNPH晶体中CCs本底值较高，为确保实验室内衍生单标的纯度及准确度，根据文献［^23^］，在衍生单标前对市售DNPH晶体进行重结晶提纯，得到高纯度DNPH晶体。

### 1.3 标准品的衍生

根据文献［^14^，^23^］衍生单标。准确称取400 mg提纯后的DNPH晶体，溶解到2 mL浓硫酸的烧杯中，加入3 mL超纯水和10 mL乙醇稀释，制备成DNPH使用液。将适量的CCs未衍生单标溶解到20 mL乙醇中，制备成CCs未衍生单标使用液，再将其缓慢加入制备好的DNPH使用液中，得到CCs单标衍生反应液（单羰基化合物与DNPH物质的量之比为2∶1，二羰基化合物与DNPH物质的量之比为1∶4），70 ℃水浴加热，充分反应30 min，随后冰水浴静置10 min，至无晶体再析出，去除上清液再向烧杯中加入30 mL乙醇，70 ℃水浴加热30 min，随后冰水浴静置10 min。重复上述结晶步骤3次，去除上清液，高纯氮气吹干晶体，装瓶避光保存，即得到高纯度CCs-DNPH衍生晶体。CCs-DNPH衍生结晶使用乙腈配制至合适质量浓度，经仪器检验无杂峰。

### 1.4 衍生混合标准工作液的配制

准确称取适量的CCs-DNPH衍生晶体至5 mL容量瓶中并用乙腈定容，配制成质量浓度为100 mg/L（以CCs计）的单标储备液，共计16种。准确移取适量的24种市售CCs-DNPH衍生混合标准储备液和16种实验室CCs-DNPH衍生单标储备液至5 mL容量瓶中并用乙腈定容，配制成质量浓度为5 000 μg/L的40种CCs-DNPH衍生混合标准使用液，于4 ℃密闭避光保存。准确移取适量的40种CCs-DNPH衍生混合标准使用液至5 mL容量瓶中并用乙腈定容，配制成质量浓度分别为2.5、5、10、25、50、100、200 μg/L的40种CCs-DNPH衍生化混合标准工作液。

### 1.5 样品采集及前处理

#### 1.5.1 样品采集

使用大气采样器采集环境空气样品，将DNPH管接到进气端，再将除臭氧小柱接到采样管的前端，在采样泵的作用下，空气样品先进入除臭氧小柱、消除臭氧，再进入DNPH管，CCs与DNPH发生衍生化反应而被采集。采样流量为1 L/min，采样时长为4 h。采样结束后，将DNPH管两端用密封帽封闭、密封避光包装，低温（<4 ℃）保存与运输，需20 d内完成前处理。

#### 1.5.2 样品前处理

在真空干燥箱中，将采样后的DNPH管在常温下用4 mL乙腈反向洗脱（乙腈自然流过DNPH管，流向与采样方向相反）至5 mL容量瓶中，并用乙腈定容。用注射器吸取洗脱液，经0.22 μm有机滤膜过滤后转移至1.5 mL棕色进样瓶中，待仪器分析。

### 1.6 分析条件

#### 1.6.1 色谱条件

色谱柱：Sepax BR-C18 （250 mm×4.6 mm， 5 μm）；流动相A：1.0 mmol/L乙酸铵水溶液；流动相B：乙腈；流速：0.5 mL/min；进样量：5 μL；系统清洗液：甲醇；柱塞杆清洗液：10%异丙醇水溶液；洗针液：50%甲醇水溶液；柱温：50 ℃。梯度洗脱程序：0~27 min，65%B~80%B；27~30 min，80%B~95%B；30~50 min，95%B；50~55 min，95%B~80%B；55~60 min，80%B~65%B。

#### 1.6.2 质谱条件

离子源：电喷雾离子源（ESI）；电离模式：负离子；扫描模式：多反应监测（MRM）模式；离子源清洗液：50%异丙醇水溶液；干燥气：高纯氮气，温度350 ℃，流量11 L/min；雾化气：高纯氮气，压力240 kPa；离子源电压：3 000 V；毛细管电压：3 500 V；碰撞气：高纯氮气，流速0.5 mL/min；碰撞池加速电压：4 V。40种CCs的保留时间和质谱参数详见表1。

**表1:** 40种CCs的质谱参数

No.	Compound	Retention time/min	Precursor ion （*m/z*）	Quantitative product ion （*m/z*）	Fragmentor/V	Collision energy/eV	Dwell time/ms
1	formaldehyde （甲醛）^*^	10.30	209.1	163.0	135	10	200
2	acetaldehyde （乙醛）^*^	12.22	223.1	163.1	135	10	200
3	2-furaldehyde （糠醛）^*^	13.89	275.0	228.0	110	5	200
4	arolein （丙烯醛）^*^	14.35	235.1	158.2	125	20	200
5	acetone （丙酮）^*^	14.74	237.1	207.3	120	25	200
6	propionaldehyde （丙醛）^*^	15.59	237.1	162.9	115	15	200
7	salicylaldehyde （水杨醛）^**^	15.91	301.0	182.0	80	5	150
8	2-butenal （丁烯醛）^*^	17.55	249.1	172.1	75	5	150
9	methacrolein （甲基丙烯醛）^*^	18.43	249.1	172.1	75	5	150
10	glyoxal （乙二醛）^**^	19.23	417.1	182.1	90	10	150
11	2-butanone （2-丁酮）^*^	19.27	251.1	152.1	115	10	150
12	butanal （丁醛）^*^	19.46	251.2	163.3	115	15	150
13	benzaldehyde （苯甲醛）^*^	20.28	285.2	163.1	75	20	200
14	isovaleraldehyde （异戊醛）^*^	23.11	265.2	152.1	80	15	200
15	2-pentanone （2-戊酮）^**^	23.54	265.0	152.0	115	20	200
16	acetophenone （苯乙酮）^**^	23.70	299.0	254.0	125	20	100
17	cyclohexanone （环己酮）^*^	23.73	277.2	247.1	95	5	200
18	methylglyoxal （甲基乙二醛）^**^	23.96	431.1	182.1	125	10	150
19	pentanal （戊醛）^*^	24.05	265.2	152.2	115	15	200
20	*p*-tolualdehyde （对甲基苯甲醛）^*^	24.36	299.2	163.1	125	10	100
21	*m*-tolualdehyde （间甲基苯甲醛）^*^	24.85	299.2	163.1	85	10	100
22	*o*-tolualdehyde （邻甲基苯甲醛）^*^	25.13	299.2	163.2	95	10	100
23	*trans*-2-hexenal （反式-2-己烯醛）^**^	27.10	277.0	163.3	75	5	100
24	methyl isobutyl ketone （甲基异丁基酮）^*^	27.53	279.2	152.2	100	15	100
25	hexanal （己醛）^*^	29.28	279.2	152.0	90	10	100
26	2，5-dimethylbenzaldehyde （2，5-二甲基苯甲醛）^*^	29.30	313.2	181.0	85	20	200
27	2-methylcyclohexanone （2-甲基环己酮）^**^	30.47	291.0	152.2	90	15	200
28	6-methyl-5-hepen-2-one （6-甲基-5-庚烯-2-酮）^**^	32.62	305.0	152.2	100	20	200
29	nopinone （诺蒎酮）^**^	33.20	317.0	152.1	105	20	200
30	2-heptanone （2-庚酮）^**^	33.99	293.0	152.2	110	5	200
31	heptanal （庚醛）^*^	34.30	293.2	163.0	85	5	200
32	isophorone （异佛尔酮）^**^	34.31	317.0	152.1	95	10	200
33	limona ketone （4-乙酰基-1-甲基-环己烯）^**^	35.07	317.0	152.1	95	10	200
34	2-octanone （2-辛酮）^**^	36.51	307.0	152.1	85	5	200
35	octanal （辛醛）^*^	36.70	307.3	163.1	75	10	200
36	nonanal （壬醛）^*^	38.96	321.3	152.0	95	15	200
37	decanal （癸醛）^*^	41.70	335.2	163.0	90	10	200
38	undecanal （十一醛）^**^	45.05	349.0	162.8	95	15	200
39	dodecanal （十二醛）^**^	49.26	363.0	163.4	90	15	200
40	tridecanal （十三醛）^**^	54.61	377.0	162.8	95	15	200

* The standards were commercial； ** The standards were homemade.

## 2 结果与讨论

### 2.1 色谱条件的优化

#### 2.1.1 色谱柱的优化

现有HPLC-MS方法最多仅可测定30余种CCs^［4，13，14，16］^，而本研究需单次进样同时测定40种CCs，对色谱柱的分离效果要求更高。因此，本研究在同一分析仪器上，采用相同的质谱条件和柱流速，依次对3根色谱柱进行分离测试：通过优化各自的最佳梯度洗脱程序，分别考察了Sepax BR-C18（250 mm×4.6 mm， 5 μm）、Agilent InfinityLab Poroshell 120 EC-C18（100 mm×4.6 mm， 2.7 μm）和Agilent Zorbax Extend-C18（250 mm×4.6 mm， 5 μm）色谱柱测定40种CCs-DNPH衍生混合标准使用液（150 μg/L）的峰形及分离效果。结果表明（见图1），Agilent InfinityLab Poroshell 120 EC-C18色谱柱（100 mm×4.6 mm， 2.7 μm）虽然具有最短的分析时间、最窄的半峰宽，但不能将离子对相同的3组目标物（2-丁酮与丁醛、对甲基苯甲醛与间甲基苯甲醛、2-辛酮与辛醛）有效分离；Agilent ZORBAX Extend-C18色谱柱（250 mm×4.6 mm， 5 μm）不能将间、对-甲基苯甲醛有效分离；而Sepax BR-C18色谱柱（250 mm×4.6 mm， 5 μm）的分析时间（60 min）适中、半峰宽较窄，结合优化的质谱条件能够较好地将所有目标物有效分离，解决了现有HPLC-MS方法中保留时间相近、离子对相同目标物难以有效分离的问题。因此选择Sepax BR-C18色谱柱（250 mm×4.6 mm， 5 μm）。

**图1:**
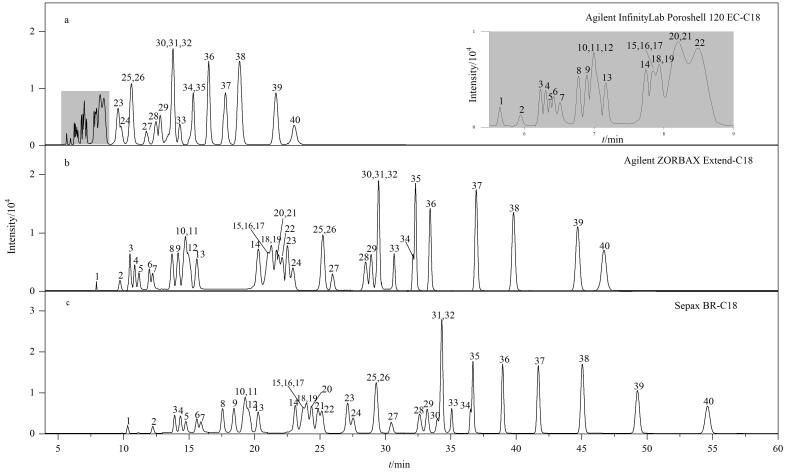
采用不同色谱柱时40种CCs-DNPH衍生物的提取离子色谱图

#### 2.1.2 流动相的优化

乙腈和甲醇同为常用流动相中的有机相。研究表明，相较于甲醇，乙腈的传质阻力更低，洗脱能力更强，进而有效降低方法用时，使得流动相基线更平稳，峰形良好，此外，CCs-DNPH衍生物在乙腈-水流动相中比在甲醇-水流动相中的灵敏度更高，重复性更好，与文献［^12^，^14^，^15^］的研究结果相符。因此选择乙腈作为有机相。

流动相添加剂可调控流动相的酸碱度，有利于在电喷雾过程中形成带电离子，乙酸铵为测定CCs-DNPH衍生物的最佳选择^［7］^。研究表明，乙酸铵难以完全溶解于乙腈中，在有机相和水相中均添加乙酸铵与仅在水相中添加乙酸铵对目标物峰面积的影响差异不大，与文献［^14^］的研究结果相符。因此选择仅在水相中添加乙酸铵。将乙酸铵浓度分别设置为0、0.5、1.0和1.5 mmol/L，考察各目标物的峰面积变化。结果表明（见图2），在0~1.0 mmol/L范围内，随着乙酸铵浓度的增加，LMW-MCs的峰面积变化不明显或略有降低，而HMW-MCs和DCs的峰面积均呈现明显的增强趋势；当浓度增至1.5 mmol/L时，各目标物的峰面积均不再增强，反而部分目标物的峰面积有所降低，可能是因为乙酸铵浓度过高与目标物离子电荷占据液滴表面产生竞争即离子抑制，反而降低了目标物的离子化效率。因此乙酸铵浓度设为1.0 mmol/L。

**图2:**
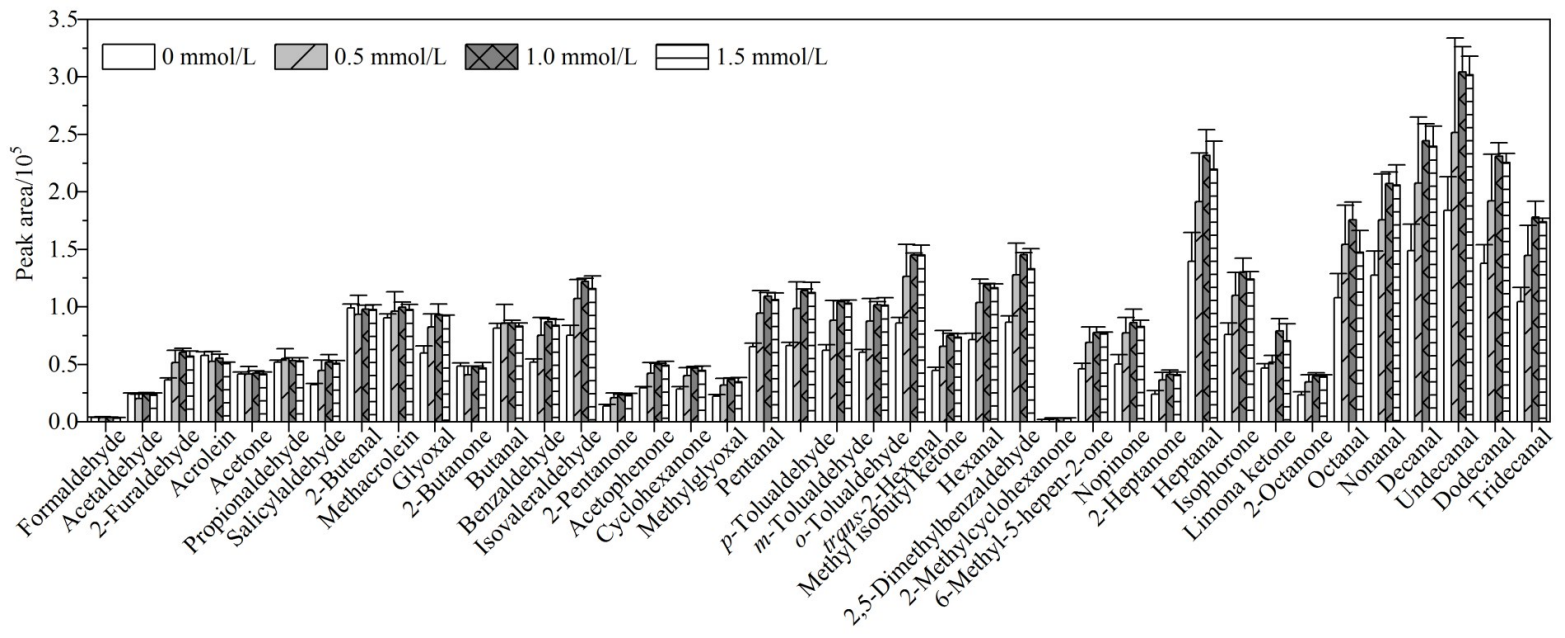
采用不同质量浓度的乙酸铵时40种CCs-DNPH衍生物的峰面积（*n*=3）

### 2.2 质谱条件的优化

CCs-DNPH衍生物偏弱酸性^［9］^，在ESI源中，正离子模式下无明显信号输出，负离子模式下会产生信号强且稳定的去质子分子离子［M-H］^-^，因此选择负离子模式检测。ESI源的离子化效率受雾化气压力、干燥气流量、干燥气温度、毛细管电压等参数的影响^［4，9，16］^。因此，采用优化后的色谱条件，在负离子模式下（使用默认的毛细管电压：3 500 V），将40种CCs-DNPH衍生混合标准使用液（150 μg/L）进行多次注射（5 μL），对雾化气压力在130~340 kPa范围内进行了评估，得出240 kPa为最佳条件。同理对ESI源的干燥气流量和温度进行了优化，最佳条件确定为11 L/min和350 ℃。所有优化后的ESI源参数值详见1.6.2节。

MRM扫描可最大限度实现各目标物的准确定性、定量^［16］^，因此选择MRM扫描模式检测。首先，采用优化后的色谱条件和ESI源参数，依次使用40种CCs-DNPH衍生单标（质量浓度均为100 μg/L）在负离子模式下进行全扫描（MS^2^ Scan）（扫描范围为*m/z* 200~500），确认各目标物的保留时间和母离子［M-H］^-^信息并做定性分析。再进行子离子扫描（Product Ion Scan），选择合适的特征子离子作为定量子离子，与母离子组成离子对。基于保留时间、离子对、优化后的离子源参数和色谱条件建立基础采集方法。最后利用Agilent MassHunter Optimizer软件，通过多次注射40种CCs-DNPH衍生混合标准使用液（150 μg/L），进一步优化质谱参数（碎裂电压和碰撞能量）：先将碎裂电压优化区间设置为30~200 V，碰撞能量优化区间设置为0~50 eV并进行粗调，再基于粗调结果缩小碎裂电压和碰撞能量的优化区间范围并进行细调。结果表明（见表1），LMW-MCs（丁烯醛和甲基丙烯醛除外）的最佳碎裂电压较高（115~135 V），LMW-MCs（2-戊酮、苯乙酮、戊醛和对甲基苯甲醛除外）的最佳碎裂电压较低（75~110 V），各目标物的最佳碰撞能量无显著规律，为5~25 eV。将Optimizer优化结果直接引入采集方法中，根据目标物的性质和保留时间，调整扫描时间段和驻留时间的分配。40种CCs-DNPH衍生混合标准液的提取离子流色谱图如图1c所示。

### 2.3 样品采集和前处理的优化

许多目标物（尤其HMW-MCs）的环境含量偏低^［7，17］^，可通过增大采样体积、降低方法检出限的方式有效检出。然而DNPH管的吸附容量有限，采样流量和采样时长对吸附效率均有影响^［6，9，23］^，为避免穿透，应给予优化。甲醛、壬醛和甲基乙二醛是环境空气中质量浓度较高、反应活性差异较大且代表性较强的3种CCs^［9，17］^，因此以三者为例来确定最佳采样条件。首先将采样时长设定为3 h，采样流量分别设置为0.6、0.8、1.0和1.2 L/min，基于穿透实验（使用2根DNPH管串联采样，将前后两根管进行相同的前处理和上机测试，计算采样后管中目标物的量与前管中相应目标物的量之比，即穿透率）^［24］^，考察3种代表性CCs的穿透率变化。结果表明（见图3），3种代表性CCs在各采样流量下均有不同程度的穿透现象发生，在0.6~1.0 L/min范围内，穿透率均较小（<8.0%）；而当流量增至1.2 L/min时，穿透率均陡增且超出达标限值（10.0%），因此采样流量确定为1.0 L/min。基于优化后的采样流量，采用相同的方式优化采样时长，综合考虑采样体积（尽量大）和穿透率（<10.0%），最佳采样时长确定为4 h。

**图3:**
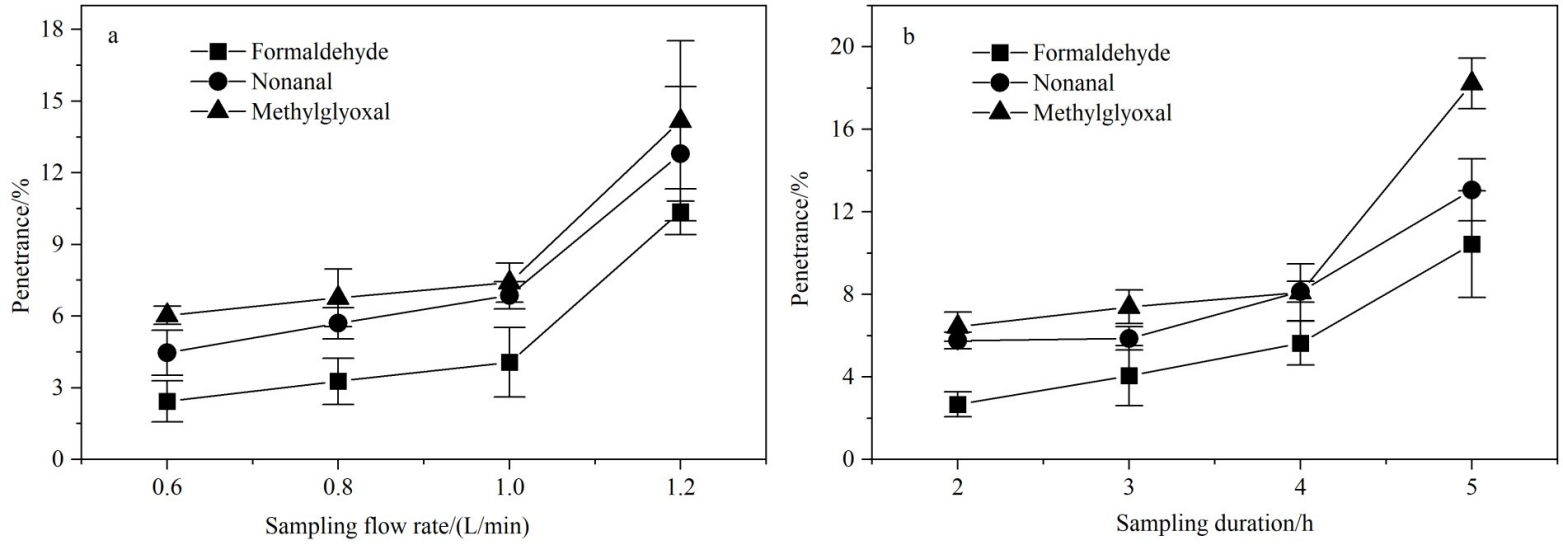
采用不同（a）采样流量和（b）采样时长时3种代表性CCs的穿透率（*n*=3）

在空白DNPH管中加入20 μL 40种CCs-DNPH衍生化混合标准使用液（5 000 μg/L），制备成DNPH加标测试管。为确保洗脱溶剂（乙腈）对DNPH管中目标物的洗脱效果，依次使用1 mL乙腈逐步洗脱DNPH加标测试管5次，分别收集洗脱液，并计算回收率（每次洗脱目标物的量扣除本底后与相应目标物的理论加标量之比）^［24］^。同样以甲醛、壬醛和甲基乙二醛为例来确定洗脱溶剂的最佳用量。结果表明（见图4），在加入1 mL时回收率均较低（5.8%~11.5%），随着洗脱溶剂用量的增加，累计回收率均显著增高；当加入4 mL时，累计回收率高达98.8%~99.5%；再继续洗脱，回收率已无显著变化。综合考虑回收率和定容量（过大会影响样品中低含量CCs的有效检出），最终选择洗脱溶剂用量为4 mL。由于优化后的洗脱溶剂用量较少，而样品采集体积较大可能会影响洗脱效率，按照上述方式，对实际样品进行5次逐步洗脱，并计算洗脱效率（每次洗脱目标物的量与5次洗脱后相应目标物的总量之比）。结果表明，洗脱溶剂用量为4 mL时，3种代表性CCs的累计洗脱效率均超过99.5%，洗脱效果良好。

**图4:**
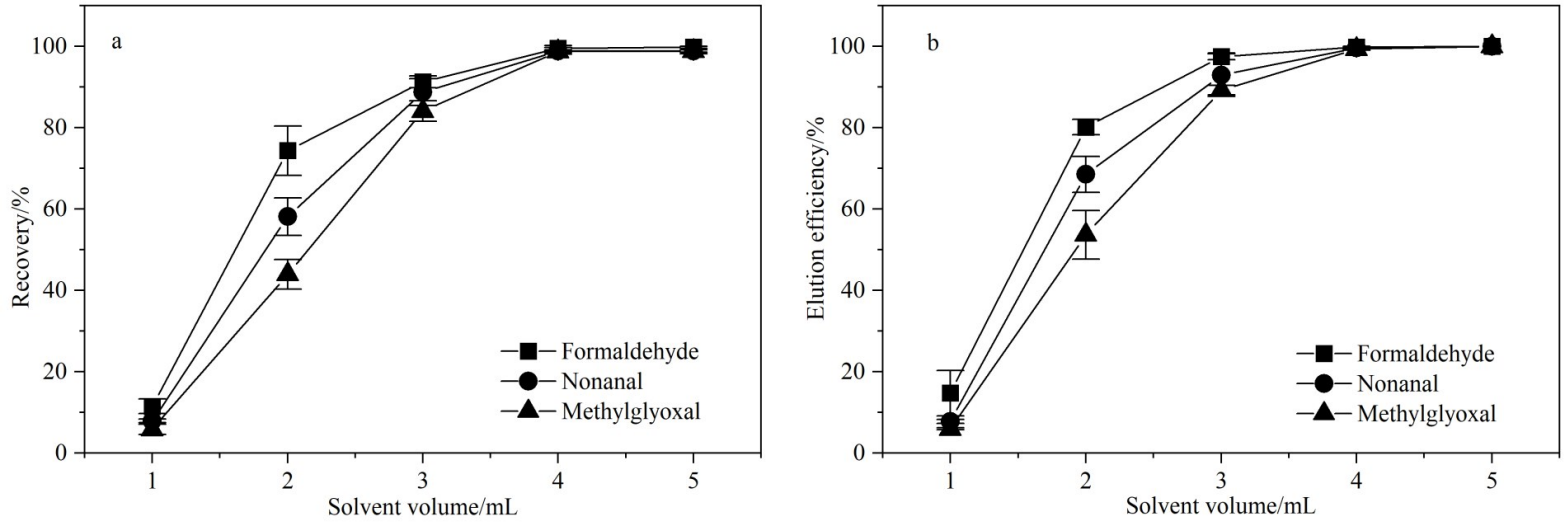
采用不同洗脱溶剂量时3种代表性CCs的（a）回收率和（b）洗脱效率（*n*=3）

### 2.4 方法学验证

#### 2.4.1 标准曲线与检出限

按照优化后的仪器工作条件测定40种CCs-DNPH衍生化混合标准工作溶液，以系列标准溶液质量浓度（*x*，μg/L）为横坐标、定量子离子峰面积（*y*）为纵坐标进行线性回归分析。结果表明（见表2），40种CCs在2.5~200 μg/L范围内具有良好的线性关系，决定系数（*R*^2^）为0.997 8~1.000 0。

**表2:** 40种CCs的回归方程、决定系数、方法检出限、方法定量限、回收率和相对标准偏差

Compound	Regression equation	*R*^2^	MDL*/*（μg/m^3^）	MQL*/*（μg/m^3^）	Recovery/%	RSD/%
Formaldehyde	*y*=3.80×10^4^ *x*+1.17×10^1^	0.9998	0.01	0.05	84.8-87.9	0.6-0.7
Acetaldehyde	*y*=1.74×10^5^ *x*+9.81×10^1^	0.9998	0.006	0.02	82.3-88.3	0.6-1.0
2-Furaldehyde	*y*=4.48×10^5^ *x*+3.42×10^2^	0.9999	0.01	0.05	75.2-75.7	2.8-3.3
Acrolein	*y*=4.73×10^5^ *x*+3.62×10^0^	0.9995	0.01	0.05	76.3-76.4	0.7-2.1
Acetone	*y*=2.73×10^5^ *x*+4.14×10^2^	0.9999	0.005	0.02	78.8-79.4	0.4-0.9
Propionaldehyde	*y*=3.58×10^5^ *x*+9.69×10^2^	0.9996	0.006	0.02	86.1-87.3	1.1-2.4
Salicylaldehyde	*y*=3.11×10^5^ *x*-3.04×10^2^	0.9995	0.005	0.02	79.6-82.5	1.2-3.4
2-Butenal	*y*=6.56×10^5^ *x*+5.66×10^2^	0.9993	0.008	0.03	80.8-83.8	1.3-2.5，
Methacrolein	*y*=6.64×10^5^ *x*+1.41×10^3^	0.9996	0.01	0.04	83.5-83.6	1.5-2.1
Glyoxal	*y*=4.57×10^5^ *x*+1.16×10^0^	0.9995	0.005	0.02	90.3-103.2	2.4-3.1
2-Butanone	*y*=3.22×10^5^ *x*+2.92×10^2^	0.9999	0.005	0.02	87.1-91.3	2.5-3.4
Butanal	*y*=5.74×10^5^ *x*+8.68×10^2^	0.9998	0.01	0.05	83.1-87.1	2.1-3.2
Benzaldehyde	*y*=5.59×10^5^ *x*+1.32×10^2^	0.9996	0.01	0.05	82.5-86.3	2.1-4.3
Isovaleraldehyde	*y*=7.97×10^5^ *x*+9.37×10^2^	0.9998	0.01	0.04	86.2-87.5	1.0-1.2
2-Pentanone	*y*=3.37×10^5^ *x*-4.67×10^2^	0.9991	0.004	0.02	87.9-103.6	1.6-2.7
Acetophenone	*y*=3.04×10^5^ *x*+6.20×10^2^	0.9998	0.01	0.04	88.8-109.9	2.6-3.3
Cyclohexanone	*y*=4.09×10^4^ *x*+1.84×10^1^	0.9998	0.009	0.04	82.5-83.4	3.5-4.6
Methylglyoxal	*y*=2.35×10^5^ *x*-5.17×10^2^	0.9993	0.02	0.07	89.2-103.1	3.4-5.1
Pentanal	*y*=6.65×10^5^ *x*+1.11×10^3^	0.9998	0.009	0.04	88.2-89.9	2.1-3.3
*p*-Tolualdehyde	*y*=7.36×10^5^ *x*-5.01×10^2^	0.9996	0.01	0.06	80.0-83.1	1.2-1.5
*m*-Tolualdehyde	*y*=6.48×10^5^ *x*-7.23×10^3^	0.9993	0.01	0.05	82.5-84.3	2.1-2.2
*o*-Tolualdehyde	*y*=6.14×10^5^ *x*-2.14×10^2^	0.9994	0.02	0.06	82.7-83.1	2.4-3.4
*trans*-2-Hexenal	*y*=9.36×10^5^ *x*-7.59×10^2^	0.9994	0.004	0.02	85.9-81.2	0.9-1.1
Methyl isobutyl ketone	*y*=4.38×10^5^ *x+*2.49×10^2^	1.0000	0.006	0.02	77.8-81.6	2.1-2.2
Hexanal	*y*=7.16×10^5^ *x+*8.34×10^2^	0.9998	0.007	0.03	92.7-88.0	1.9-2.1
2，5-Dimethylbenzaldehyde	*y*=8.75×10^5^ *x*-6.49×10^2^	0.9995	0.01	0.04	86.0-103.2	1.7-1.8
2-Methylcyclohexanone	*y*=1.56×10^4^ *x*-1.18×10^1^	0.9992	0.02	0.06	118.1-119.0	1.5-1.8
6-Methyl-5-hepen-2-one	*y*=3.52×10^5^ *x*-3.94×10^2^	0.9986	0.004	0.02	92.9-99.6	2.3-3.1
Nopinone	*y*=5.52×10^5^ *x*-6.48×10^2^	0.9993	0.002	0.01	78.3-78.7	3.6-4.2
2-Heptanone	*y*=4.39×10^5^ *x+*1.97×10^2^	0.9994	0.02	0.08	83.5-109.3	2.8-3.4
Heptanal	*y*=1.47×10^6^ *x+*1.83×10^3^	0.9997	0.01	0.04	87.6-89.3	2.1-3.5
Isophorone	*y*=7.92×10^5^ *x*-7.23×10^2^	0.9991	0.001	0.005	80.7-81.4	3.1-3.6
Limona ketone	*y*=5.01×10^4^ *x*+4.43×10^1^	0.9978	0.009	0.04	113.9-116.0	3.7-4.1
2-Octanone	*y*=6.82×10^5^ *x*-6.68×10^2^	0.9989	0.003	0.01	90.8-94.8	3.5-4.2
Octanal	*y*=2.06×10^6^ *x*+2.79×10^3^	0.9997	0.02	0.11	88.9-95.6	3.7-4.2
Nonanal	*y*=2.21×10^6^ *x*+2.30×10^3^	0.9997	0.03	0.11	88.7-100.3	2.3-3.7
Decanal	*y*=2.46×10^6^ *x*+2.33×10^3^	0.9997	0.03	0.11	85.4-91.1	3.4-4.6
Undecanal	*y*=2.30×10^6^ *x*-3.56×10^3^	0.9991	0.003	0.01	85.9-88.2	2.8-3.2
Dodecanal	*y*=2.20×10^6^ *x*-3.43×10^3^	0.9989	0.002	0.01	89.5-90.1	2.7-4.1
Tridecanal	*y*=5.37×10^5^ *x*-3.52×10^2^	0.9994	0.03	0.12	91.0-106.0	2.6-3.4

*y*： peak area； *x*： mass concentration， μg/L.

取适量的40种CCs-DNPH衍生化混合标准使用液配制成质量浓度为2.5 μg/L的混合标准测试液，重复测定7次，按照采样体积为240 L、定容量为5 mL计算测试结果。根据方法检出限（MDL）计算公式：MDL=*t*_（_*_n_*_-1，0.99）_×*S*（*S*为*n*次平行测定的标准偏差，当*n*=7时，*t*取值3.143）计算MDL，4倍MDL作为方法定量限（MQL）^［24］^。结果表明（见表2），40种目标物的MDL为0.001~0.03 μg/m^3^，MQL为0.005~0.12 μg/m^3^。本方法中HMW-MCs和DCs的MDL（0.001~0.03 μg/m^3^和0.005~0.02 μg/m^3^）远低于HPLC-UV法（HMW-MCs的MDL为0.2~0.7 μg/m^3 ［6，13］^，DCs的MDL为0.03~0.04 μg/m^3 ［21］^）、GC-MS法（HMW-MCs的MDL为0.05~0.2 μg/m^3 ［11］^，DCs无相关报道）等常规方法，有效解决了实际样品中低质量浓度DCs和HMW-MCs检测困难的问题。

#### 2.4.2 回收率与精密度

取适量的40种CCs-DNPH衍生混合标准使用液，添加到干净未使用的DNPH管中，制备成低（0.05 μg）、中（0.1 μg）和高（0.2 μg）3个加标水平的空白加标管。并按照实际样品的前处理方式进行洗脱、定容，重复测定7次，定量结果扣除空白。结果表明（见表2），40种目标物的回收率为75.2%～119.0%，相对标准偏差（RSD）为0.4%～5.1%。质控指标均优于HJ 683-2014^［18］^要求，满足环境空气中CCs的测定需求。

### 2.5 实际样品测定

2021年9月23日在珠三角5个观测站点同一时段（12：00~16：00）各采集1个环境空气常规样品和1个平行样品，按照本文建立的方法进行测定。结果表明，虽然本方法中8种CCs的MDL（≥0.02 μg/m^3^，见表2）相对较高，但远低于其在实际样品中的含量，未对准确定量产生影响。整体上，除糠醛和异佛尔酮2种HMW-MCs外，其余38种CCs均可有效检出。平行样测定结果的相对偏差为0.2%~18.2%，表明本方法测定重复性较好、定量结果准确可信。

由图5可知，采样期间，总CCs的含量为（50.90±17.31） μg/m^3^，其中含量最高的6种CCs依次为甲醛（8.23±0.75） μg/m^3^、乙醛（6.58±3.74） μg/m^3^、丙酮（5.77±3.61） μg/m^3^、壬醛（4.28±1.21） μg/m^3^、己醛（3.83±2.98） μg/m^3^和甲基乙二醛（3.34±1.45） μg/m^3^，均远高于其MQL，并且甲醛、乙醛和壬醛的含量在各站点均列前六；与2021‒2022年珠三角地区相关研究结果基本相符^［1，8］^。将CCs划分为LMW-MCs、HMW-MCs和DCs三类，其中LMW-MCs的含量最高（以甲醛、乙醛和丙酮为主），占总CCs的48.0%；HMW-MCs的含量次之（以壬醛、己醛和庚醛为主），占总CCs的43.3%；DCs的含量最低，仅占总CCs的8.7%，但2种DCs的含量在各站点均远高于其MQL。

**图5:**
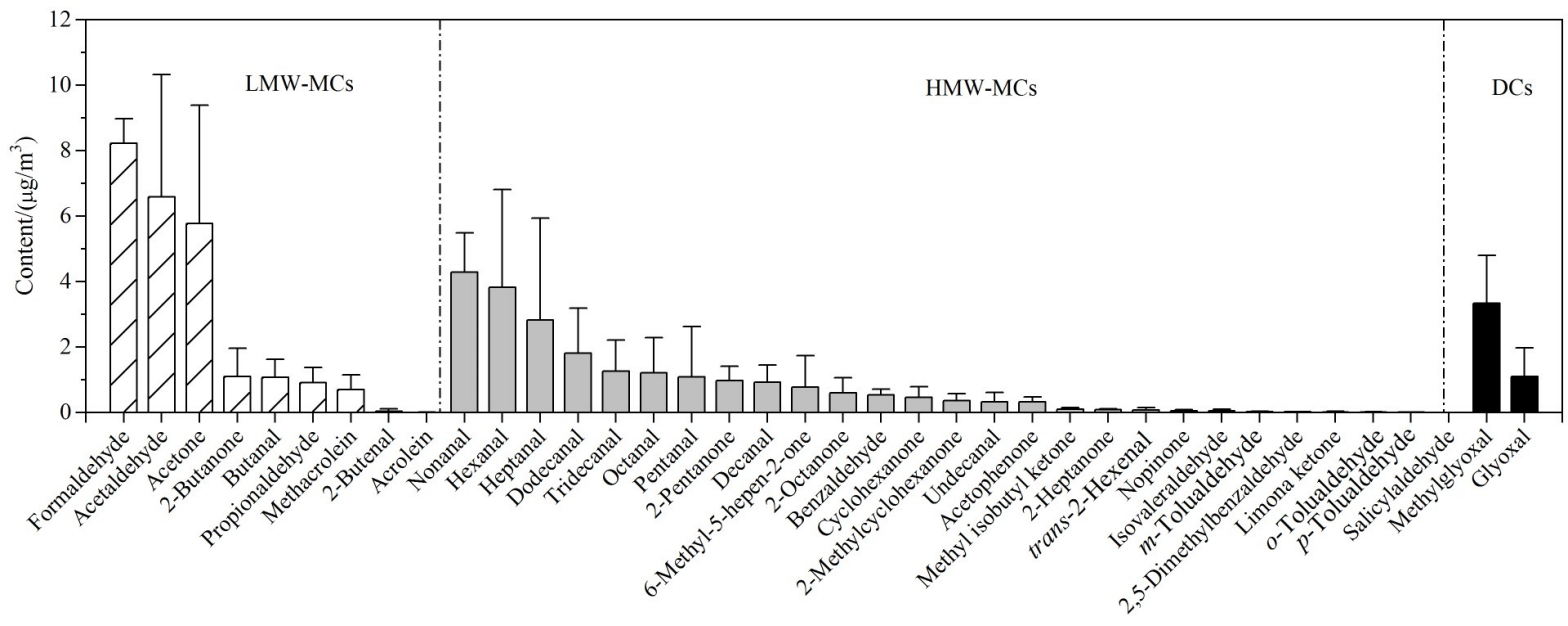
实际样品中CCs含量分布（*n*=5）

## 3 结论

本研究使用DNPH管采样，乙腈洗脱、定容，HPLC-ESI-MS/MS分析，经过方法优化，建立了适用于环境空气中40种CCs的分析方法。相较于现有方法，本方法增加了目标物中DCs和HMW-MCs的种类数，有效解决了现有方法测定CCs种类少的问题；改善了保留时间相近、离子对相同的目标物的分离效果。整体上，本方法简便快速，稳定灵敏，高效准确，可完全满足实际样品的准确检测需求，为进一步研究大气氧化机制，制定污染控制策略，提供更为全面、可靠的CCs信息及技术支撑。
